# Awareness and Perceived Risk Factors of Chronic Kidney Disease Among Patients with Diabetes in the Northern Borders of Saudi Arabia: Implications for a Strategic Monitoring and Management Plan

**DOI:** 10.3390/diseases14020074

**Published:** 2026-02-16

**Authors:** Safya E. Esmaeel, Altaf Saleh Mahdi Alanazi Alnzi, Nouf Mofareh Mulahed Alanazi, Rose Dahi Khamis Alanazi, Amal Mohammed Shahi Alruwaili, Areeb Rawaf Mohammed Alanzi, Ahad Wadi Alnagzi Alanazi, Yousef Wasmi Alenezi, Ahmed Saleh Alanazi, Rimas Khalid A. Alanazi, Baraah Abu Alsel, Eslam K. Fahmy, Manal S. Fawzy

**Affiliations:** 1 Department of Physiology, College of Medicine, Northern Border University, Arar 91431, Saudi Arabia; safya.ebraheem@nbu.edu.sa (S.E.E.); eslam.kamal@nbu.edu.sa (E.K.F.); 2 College of Medicine, Northern Border University, Arar 91431, Saudi Arabia; st202310948@stu.nbu.edu.sa (A.S.M.A.A.); st202309323@stu.nbu.edu.sa (N.M.M.A.); st202310840@stu.nbu.edu.sa (R.D.K.A.); st202310935@stu.nbu.edu.sa (A.M.S.A.); st202310534@stu.nbu.edu.sa (A.R.M.A.); st202310357@stu.nbu.edu.sa (A.W.A.A.); st202000218@stu.nbu.edu.sa (Y.W.A.); st202000221@stu.nbu.edu.sa (A.S.A.); st202101048@stu.nbu.edu.sa (R.K.A.A.); 3 Medical Sciences and Preparatory Year Department, North Private College of Nursing, Arar 73312, Saudi Arabia; baraahsel@nec.edu.sa; 4 Center for Health Research, Northern Border University, Arar 73213, Saudi Arabia

**Keywords:** awareness, chronic kidney disease, diabetes mellitus, northern border region, prevalence, Saudi Arabia

## Abstract

**Background/Objectives**: Chronic kidney disease (CKD) poses a significant health burden for individuals with diabetes mellitus, increasing morbidity and mortality. Understanding CKD and its risk factors is essential for early detection, effective management, and prevention of complications. This study aimed to assess CKD awareness as the primary outcome and to explore self-reported CKD prevalence and associated factors as secondary outcomes among patients with diabetes in the Northern Border Region, Saudi Arabia. **Methods**: A cross-sectional survey was conducted among 389 adults with a self-reported physician diagnosis of diabetes in the specified region, using a validated, self-administered online questionnaire. Data were analyzed to evaluate CKD awareness and identify perceived risk factors and factors associated with self-reported CKD. **Results**: Of the participants, 182 (46.8%) demonstrated good awareness of CKD, while the self-reported prevalence of CKD was 83 (21.3%). Males and unmarried participants were more likely to have good CKD awareness (*p* = 0.008 and 0.009, respectively). Significant associations were observed between self-reported CKD prevalence and age, sex, type of diabetes, family history of kidney disease, and comorbidities (all *p* < 0.05). Multivariate logistic regression showed that hypertension was strongly associated with self-reported CKD [aOR = 5.77; 95% CI: 3.12–10.67; *p* < 0.001], as was heart disease [aOR = 4.21; 95% CI: 1.35–13.13; *p* = 0.013]. **Conclusions**: Patients with diabetes in this region exhibited moderate awareness of CKD, with higher awareness among males and unmarried individuals. Hypertension and cardiac disease were significantly associated with self-reported CKD. These findings underscore the importance of targeted education, routine evidence-based screening, and structured management strategies for CKD within diabetes care and provide a basis for a regional strategic plan to strengthen CKD monitoring among patients with diabetes.

## 1. Introduction

Chronic kidney disease (CKD) is an escalating global health challenge, characterized by the gradual decline of renal function and a reduced capacity to maintain metabolic and fluid balance [1]. As noncommunicable diseases have surpassed infectious diseases and malnutrition as leading global causes of death, CKD has emerged as a major contributor to worldwide morbidity and mortality [2]. Early CKD is frequently underdiagnosed because it often remains asymptomatic, with only subtle indicators such as albuminuria or mildly reduced estimated glomerular filtration rate (eGFR); without prompt detection and intervention, many patients progress to end-stage renal disease, necessitating dialysis or transplantation [3]. Importantly, early identification and proactive management can significantly reduce the risk of adverse outcomes and slow CKD progression, particularly when kidney damage is detected in its initial stages [4]. Nevertheless, insufficient awareness and knowledge of CKD and its multifactorial risk factors among both patients and providers often result in delayed diagnosis, with many individuals presenting at advanced, irreversible stages of disease [5].

Diabetes mellitus (DM), driven by sedentary lifestyles, suboptimal dietary habits, and other modifiable risk factors, has become the world’s fastest-rising chronic health condition, with type 2 diabetes mellitus (T2DM) accounting for approximately 90% of all cases [6]. The Middle East and North Africa region currently ranks among the highest globally in diabetes prevalence, further compounding the regional burden of chronic diseases [7]. This is particularly concerning given that diabetes and hypertension are the primary etiological factors for CKD, and that diabetic kidney disease often progresses without pain or overt urinary symptoms during stages 1–3, leading many patients to assume their kidneys are healthy if they “feel well” and their glycemic and blood pressure control appear satisfactory [8]. International guidelines, including consensus from the “American Diabetes Association (ADA)” and “Kidney Disease: Improving Global Outcomes (KDIGO),” now recommend at least annual CKD screening in people with diabetes using a dual approach: serum creatinine–based eGFR to assess filtration and urine albumin-to-creatinine ratio (UACR) to detect albuminuria, alongside stringent blood pressure targets (e.g., <130/80 mmHg) to preserve kidney function [4,5]. However, despite these recommendations, UACR testing is often underutilized in routine diabetes care, and CKD is sometimes considered a “downstream” complication, contributing to therapeutic inertia and missed opportunities for timely initiation of kidney-protective therapies [9].

In addition to diabetes and hypertension, other modifiable risk factors, including smoking, obesity, dyslipidemia, and metabolic abnormalities, further contribute to CKD risk and progression [10]. The reported prevalence of CKD among individuals with T2DM varies widely worldwide, ranging from about one-quarter to over four-fifths, reflecting both population heterogeneity and differences in diagnostic criteria, screening practices (eGFR alone versus combined eGFR and UACR), and study methodology [11]. Evidence from multiple countries, including high- and middle-income settings, indicates that diabetic kidney disease is common but often poorly recognized, particularly in its early stages, among people with diabetes, and that CKD awareness in this group remains suboptimal despite the high burden of disease.

Recent work from diverse regions (e.g., Saudi Arabia, Europe, Africa, and Asia) has further highlighted the gap between recommended screening and real-world practice, with many patients not receiving regular eGFR and UACR testing, and only a limited understanding of kidney-related complications of diabetes [12,13,14,15,16,17,18,19,20,21,22].

Given the silent progression and severe long-term consequences associated with CKD in people living with diabetes, CKD awareness among patients with diabetes has emerged as a key public health concern and is the central focus of the present study [23,24]. Improving disease awareness in this high-risk group is critical for both prevention and timely intervention [25]. A robust understanding of current awareness levels, perceived risk factors, and knowledge gaps among patients with diabetes can inform the development of targeted educational materials, systematic screening protocols (including routine eGFR and UACR testing), and early intervention strategies [26]. Such information can support the design of structured monitoring pathways within diabetes care and help reduce therapeutic inertia regarding kidney-protective therapies, ultimately decreasing the public health burden of CKD [27,28].

Therefore, this study was designed to assess CKD awareness and to identify associated risk factors among patients with diabetes mellitus in the Northern Border Region of Saudi Arabia. By situating our work within the growing international literature on CKD and awareness of diabetic nephropathy, including studies from Saudi Arabia, Europe, Africa, and Asia that have examined knowledge, attitudes, and practices in diabetic populations [12,13,14,15,16,17,18,19,20,21,22], we aim to add region-specific data from an understudied area. By elucidating current knowledge and beliefs about kidney function testing, perceived susceptibility, and the role of comorbidities, and by identifying existing gaps within this at-risk demographic, our findings aim to provide actionable insights for clinicians, policymakers, and public health practitioners seeking to strengthen CKD monitoring and complication-focused education within diabetes care, and to align regional practice with contemporary ADA/KDIGO guidance in addressing the growing challenge of CKD in this region.

## 2. Materials and Methods

### 2.1. Study Design and Setting

This study employed a cross-sectional design to assess awareness of and perceived risk factors for CKD among adult patients with diabetes mellitus in the Northern Border Region of Saudi Arabia. The region is served primarily by government hospitals, diabetes and endocrine clinics, and a network of primary healthcare centers that provide long-term follow-up and medication for patients with diabetes, supplemented by community-based outreach campaigns (https://saudipedia.com/en/northern-borders-province), accessed 2 February 2026. According to recent regional estimates from the General Authority for Statistics (GASTAT; https://www.stats.gov.sa/en/home) (accessed 2 February 2026) and regional health studies, this region has an estimated adult population of over 370,000. In this region, studies have indicated a high prevalence of chronic conditions [29], with diabetes mellitus among the most prominent, reported in regional studies to affect approximately 17–23% of the adult population in high-burden areas of the Kingdom [30,31]. Our sample of 389 participants, therefore, represents a modest but informative subset of the regional diabetic population. Data collection was conducted from October 2024 to March 2025, ensuring inclusion of participants from both urban and rural areas to enhance representativeness.

### 2.2. Study Population and Eligibility Criteria

Eligible participants were adults aged 18 years or older, of any gender, with a self-reported physician diagnosis of diabetes mellitus (type 1 or type 2). Only permanent residents of the Northern Border Region who were able to provide informed consent and complete the questionnaire independently were included. Exclusion criteria included individuals under 18 years of age, those residing outside the region, individuals without a diagnosis of diabetes, individuals unable to read or comprehend the survey, and those who declined to participate at the consent stage. In the questionnaire, diabetes type (type 1, type 2, or “I do not know”) was self-reported by participants and was not verified against medical records, as reflected in the 56.6% who selected “I do not know”; this limitation and the potential for misclassification are acknowledged in the Section 4.1.

### 2.3. Sample Strategy and Sample Size Determination

Recruitment employed a convenience sampling approach, combined with purposive outreach at key healthcare facilities and community events, to maximize diversity and minimize sampling bias. The minimum sample size was calculated using the Raosoft sample size calculator, based on a 95% confidence level, a 5% margin of error, and an assumed response distribution of 50%. This calculation indicated a minimum of 385 participants was required to achieve adequate statistical power to detect relevant associations. Ultimately, 389 eligible patients with diabetes completed the survey.

Participants were recruited consecutively from outpatient diabetes clinics in government hospitals, affiliated primary healthcare centers, and community outreach events in the region. Invitation links to the online questionnaire were distributed by healthcare/university staff and coauthors. Individuals who declined participation did so mainly due to a lack of time or limited interest, and refusals were noted qualitatively but not systematically counted.

### 2.4. Study Instrument and Data Collection Procedures

Data were collected using a structured, self-administered Arabic questionnaire, the predominant language in the region. The questionnaire was based on previously validated instruments [32,33,34]. A multidisciplinary panel of endocrinologists, nephrologists, public health specialists, and survey methodologists reviewed the content for cultural and linguistic appropriateness, as well as content and construct validity.

A pilot study was conducted with ten randomly selected patients with diabetes to assess clarity, internal consistency, and time to completion. Feedback resulted in minor revisions to the wording and layout of the item. The final version of the questionnaire was delivered electronically via a secure online platform, facilitating broad access and ensuring respondent anonymity. Assistance was provided by trained healthcare personnel to individuals with limited digital literacy, with care taken to avoid influencing their responses.

The questionnaire comprised four main domains: (1) Sociodemographic characteristics: age, gender, marital status, education level, nationality, and residential location (urban/rural). (2) Medical and diabetes history: type and duration of diabetes, current treatment regimen, comorbidities (e.g., hypertension, dyslipidemia, coronary artery disease), and history of kidney disease. (3) CKD awareness: ten items assessing knowledge of CKD symptoms, risk factors, disease progression, complications, management options, and trusted sources of health information. (4) Attitudes and beliefs: eight dichotomous (yes/no) items exploring general perceptions, perceived susceptibility, perceived severity, and behavioral intentions regarding CKD prevention. It is worth noting that CKD status was assessed by self-report (“Are you suffering from chronic kidney disease (CKD)?”) rather than by biochemical criteria such as eGFR or albuminuria, and no laboratory data were collected.

Participant confidentiality was safeguarded throughout, with all data de-identified before analysis.

### 2.5. Validation and Reliability Assessment

Content validity was achieved through expert review, and internal consistency of knowledge items was evaluated in the pilot phase using Cronbach’s alpha. Minor wording changes were made to improve clarity, and a small number of ambiguous items were refined before the main survey, resulting in an internally consistent final instrument.

### 2.6. Scoring System

The questionnaire assessed CKD awareness using a composite scoring approach adapted from established literature on knowledge, attitudes, and practices towards CKD prevention and early detection [13,14,15]. The instrument (Supplementary File) consisted of three principal domains: knowledge (Section SC, items 1–9), preventive attitudes and beliefs (Section SD, items 3–8), and preventive practices (Section SD, items 4 and 6). Each knowledge and attitude item was scored as 1 for a correct/positive response (“Yes” to accurate statements or “No” to negative items) and 0 for incorrect or “I don’t know” responses, resulting in a maximum knowledge score of 9 and a maximum attitude score of 6. The preventive practice domain comprised two items, each scored similarly, for a maximum practice score of 2. For each subdomain, cumulative scores were calculated and expressed as percentages of the maximum possible. Awareness and each subdomain were categorized as “poor” or “good” at a cutoff level of 60% of the total as per validated classification schemes [13,14,15]. This scoring allows for overall analysis of CKD awareness and its key behavioral components, facilitating targeted recommendations for education and intervention.

### 2.7. Statistical Analysis

Data were analyzed using IBM SPSS Statistics, version 26 (IBM Corp., Armonk, NY, USA). Descriptive statistics included means and standard deviations for continuous variables and frequencies and percentages for categorical variables.

Bivariate associations between participant characteristics and CKD awareness were examined using the Chi-square (χ^2^) test or Fisher’s exact test, as appropriate. For multivariable logistic regression models, candidate associated factors were selected a priori based on clinical and epidemiological relevance (age, sex, diabetes type, family history of kidney disease, and major comorbidities) and on evidence from previous CKD literature, rather than solely on bivariable *p*-values. All prespecified variables were retained in the final models to allow consistent adjustment for key confounders. Adjusted odds ratios (aOR) with 95% confidence intervals (CI) were reported. All statistical tests were two-tailed, and *p*-values < 0.05 were considered statistically significant.

### 2.8. Ethical Considerations

The study was conducted following the principles of the Declaration of Helsinki and with approval from the Local Bioethics Committee at Northern Border University (HAP-09-A-043; Reference No. 109/24/H, dated 29 September 2024). All participants received clear and accessible information about the study’s aims, procedures, voluntary participation, and confidentiality protections. Informed consent was obtained electronically before survey participation. No personal identifiers were collected, and all responses were anonymized to ensure privacy.

## 3. Results

The study included 389 patients with diabetes. Over 50% of participants (203, 52.2%) were aged 18–25 years, with the overwhelming majority being Saudi nationals (371, 95.4%) and a significant majority being female (303, 77.9%). Approximately 259 (66.6%) participants held a university degree or higher, and nearly half, 191 (49.5%), were unmarried. Furthermore, 272 (69.9%) of the total reside in Arar city (i.e., the capital of the Northern Border region of Saudi Arabia) (Table 1).

Table 2 summarizes the medical characteristics of the study participants. Among the 389 individuals surveyed, 124 (31.9%) had type 1 diabetes, while 45 (11.6%) had type 2 diabetes. The majority of participants (239, 61.4%) reported a duration of diabetes of less than 5 years, and over half (217, 55.8%) were receiving oral antidiabetic medications. The self-reported prevalence of CKD within the cohort was 83 (21.3%). Additionally, 104 participants (26.7%) reported a family history of CKD. The most commonly reported comorbid conditions were hypertension (98 participants, 25.2%), heart complications (20, 5.1%), eye complications (18, 4.6%), and neuropathy (16, 4.1%).

Chronic kidney disease awareness among the participants is displayed in Table 3. Most of the participants were aware that kidney failure can be fatal unless treated with dialysis or a kidney transplant (247, 63.5%) and that CKD reduces the kidneys’ ability to remove toxins from the bloodstream (224, 62.7%). Furthermore, a considerable proportion, 236 (60.7%), were aware that diabetes can be a significant factor in chronic kidney disease development and can progress to end-stage kidney failure (236, 60.7%). The most common sources of information about chronic kidney disease were doctors (154, 39.6%) and the internet (103, 26.5%).

Table 4 displays participants’ opinions and beliefs regarding CKD. Most respondents reported that their blood sugar (327 participants, 84.1%) and blood pressure (312 participants, 80.2%) were well controlled and within healthy ranges. A substantial majority (317, 81.5%) indicated they would seek care at a health center if they developed symptoms suggestive of kidney disease. Furthermore, most participants recognized the importance of early detection, with 307 (78.9%) affirming that early identification of CKD is crucial to slowing disease progression. Additionally, 301 participants (77.4%) considered CKD to be a serious, life-threatening condition.

**Figure 1:**
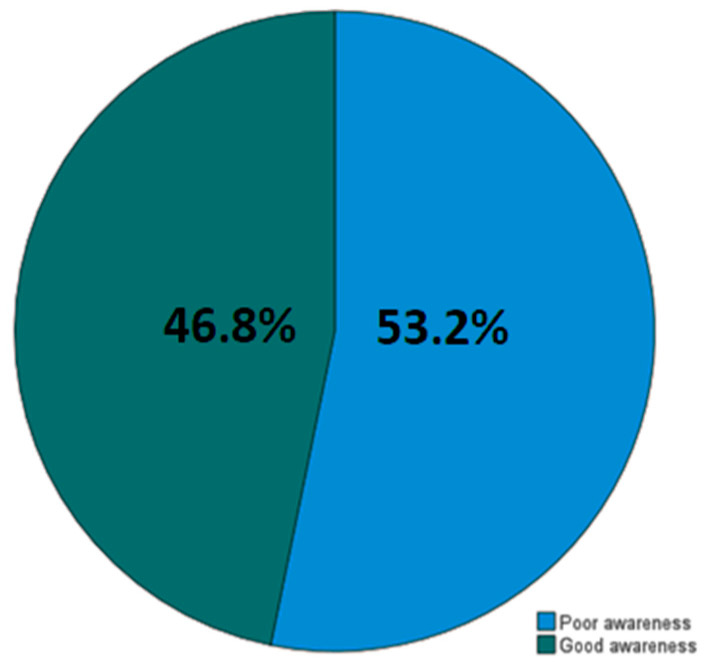
Awareness of chronic kidney disease (CKD) in the present cohort.

Among study participants, 182 (46.8%) had good awareness for CKD, while the majority, 207 (53.2%), had poor awareness (Figure 1).

According to the findings (Table 5), there are significant associations of sex and marital status with CKD awareness. Patients who are male and single are more likely to have a high level of CKD awareness (*p* = 0.008, *p* = 0.009).

There were significant associations between age, sex, type of diabetes, family history of CKD, comorbidities, and self-reported prevalence of CKD (*p* = 0.033, *p* < 0.001, *p* < 0.001, *p* < 0.001, *p* < 0.001, respectively). Self-reported CKD was more prevalent in male patients, those older than 46 years, those with type 1 diabetes, those with a family history of kidney disease, and those with high blood pressure. However, no statistically significant association was found between nationality, diabetes duration, type of diabetes treatment, and the prevalence of self-reported CKD (Table 6).

In the multivariable model, high blood pressure and heart problems remained most strongly associated with self-reported CKD, with adjusted odds ratios of 5.77 and 4.21, respectively (Table 7). Diabetes type and family history of kidney disease also showed statistically significant associations with self-reported CKD (aOR 0.33 and 0.19, respectively), indicating that CKD status differed systematically across these categories; however, the direction of these associations should be interpreted with caution, given the self-reported nature of these variables and the presence of an ‘I do not know’ category for diabetes type.

## 4. Discussion

Diabetes mellitus continues to rise globally, imposing a significant health burden due to its chronic complications, including CKD [35]. While previous studies have indicated varying levels of CKD awareness among patients with diabetes, there is a notable paucity of data from Saudi Arabia, particularly from the Northern Border region [28,34]. This study aimed to address this gap by evaluating CKD awareness and perceived risk factors among patients with diabetes in this underrepresented region.

Our findings reveal that 46.8% of patients with diabetes demonstrated good awareness of CKD, a proportion higher than reported in some Saudi and international studies [36,37], but comparable to findings from Jordan [32]. Similar to recent studies from Ethiopia, India, Malaysia, and Croatia, which have documented low to moderate levels of awareness of CKD or diabetic nephropathy among patients with diabetes, our results underscore the persistent gap between the high burden of diabetic kidney disease and patients’ understanding of it [16,18,19,20,22,38]. Studies from Palestine and other regions further show that better diabetes-related knowledge is associated with higher kidney disease knowledge and more favorable attitudes and practices, reinforcing the importance of integrating CKD education into diabetes care [17,39,40]. However, it is worth noting that our use of convenience sampling, with a predominantly young and highly educated sample, may have contributed to the relatively higher awareness observed compared with some other settings, and these figures should therefore be interpreted with caution as cohort-specific rather than fully representative of all patients with diabetes in the region.

Doctors and the internet emerged as the leading sources of CKD information, cited by 39.6% and 26.5% of participants, respectively. This highlights the critical role of healthcare professionals in patient education and reflects the increasing reliance on digital resources. Notably, male participants were more likely to have good CKD awareness than female participants, whereas married and divorced individuals showed a higher proportion of good awareness than single participants, who more frequently had poor awareness, aligning with the findings of Sa’adeh et al. [33]. This suggests that sociodemographic factors influence health literacy and should be considered when designing targeted interventions, particularly for women and married individuals who demonstrated relatively lower awareness.

The self-reported prevalence of CKD in our diabetic cohort was 21.3%, which is lower than the 43% reported by Al-Rubeaan et al. in Saudi Arabia [41] and 42.9% by Flores et al. in El Salvador [42]. Such discrepancies may be attributed to differences in study populations, diagnostic criteria (self-report versus biochemical assessment), and access to healthcare. Still, the current identified prevalence may not accurately reflect the true prevalence among all patients with diabetes in this region, given the non-probability sampling frame and reliance on self-report (without confirmatory tests as eGFR or albuminuria), which can introduce both selection and information bias.

Notably, a substantial proportion of participants (26.7%) reported a family history of kidney disease, and comorbidities such as hypertension (25.2%) and heart problems (5.1%) were common. The current results are consistent with previous research identifying hypertension, diabetes, and family history as key risk factors for CKD [43]. Univariate and multivariable analyses indicated that hypertension had the strongest association with self-reported CKD among patients with diabetes, followed by heart disease. These findings align with global evidence that highlights the synergistic effect of hypertension and diabetes on CKD risk and cardiovascular outcomes [44]. The high prevalence of comorbidities and the strong association with self-reported CKD emphasize the need for integrated management approaches in diabetic care, as recommended by recent ADA/KDIGO and national consensus reports [4,45]. Because hypertension and heart disease are closely related to each other and to diabetes, some degree of collinearity between associated cardiometabolic diseases cannot be excluded; however, the direction and magnitude of the main associations were consistent with prior evidence.

Encouragingly, most participants demonstrated positive health-seeking behaviors: 81.5% indicated they would seek medical attention if symptomatic, and 78.9% recognized the importance of early detection. However, only 54.8% reported undergoing routine annual kidney function testing, despite international recommendations calling for at least annual assessment of eGFR and albuminuria in all patients with diabetes. This discrepancy between knowledge and practice, also reported in other Saudi and regional studies, suggests missed opportunities for early detection and risk stratification in primary care and diabetes clinics [14,46,47].

### 4.1. Study Limitations

This study’s cross-sectional design limits causal inference. Accordingly, all identified relationships should be interpreted as associations rather than causal effects. Reliance on self-reported data may introduce recall and social desirability bias, particularly regarding CKD diagnosis and annual screening practices. In addition, both diabetes type and CKD status were based on self-report rather than medical records or laboratory data, with more than half of participants indicating that they did not know their diabetes type; this may have introduced misclassification and affected the reported prevalence and some of the observed associations, so these results should be interpreted with caution. Additionally, the sample was predominantly young, female, and highly educated, which may affect the generalizability of findings to other regions or demographic groups. Furthermore, recruitment relied on a convenience sampling strategy from healthcare facilities and community outreach sites, which may have led to overrepresentation of more health-engaged individuals; therefore, the estimated prevalence of self-reported CKD and the proportion with “good” awareness should be interpreted as cohort-specific rather than population-level figures. Moreover, the use of dichotomous (yes/no/I do not know) items for knowledge and attitudes may have restricted the granularity of responses compared with multi-point Likert scales, and future studies could adopt Likert-type measures to capture more nuanced gradients of CKD-related knowledge and beliefs. Future research using probability-based or registry-linked sampling frames, combined with laboratory-confirmed CKD measures and longitudinal follow-up, is needed to provide more generalizable estimates and to monitor trends in awareness and disease burden over time.

### 4.2. Implications for CKD Monitoring and a Hypothetical Strategic Plan Within Diabetes Care

The converging findings of moderate CKD awareness, substantial comorbidity burden, and incomplete uptake of annual kidney function testing in this diabetic cohort have direct implications for clinical practice and health system planning in the Northern Border region. In line with international and national guidance, there is a clear need to operationalize routine CKD monitoring and integrated risk-factor management within the diabetes services [48,49,50].

Based on our results and current guidelines, we conceptually propose a phased, theory-driven strategic plan to strengthen CKD monitoring and complication-focused education in diabetes care in the region. At the patient level, this includes targeted education modules prioritizing groups with lower awareness (e.g., females and married patients), emphasizing the silent nature of CKD, the role of hypertension and heart disease, and the importance of regular kidney function testing even in the absence of symptoms. At the clinic level, diabetes clinics should systematically implement annual eGFR and albuminuria assessment for all patients with diabetes, with more frequent monitoring for those with hypertension, cardiovascular disease, or a family history of kidney disease, and embed simple risk-stratification tools in routine workflows. At the system level, the introduction of CKD-related quality indicators (e.g., the proportion of diabetic patients receiving annual CKD screening, appropriately staging, and being referred to nephrology where indicated) could support audit, feedback, and continuous improvement in line with emerging Saudi consensus recommendations [13,14,45,47,51].

**Figure 2:**
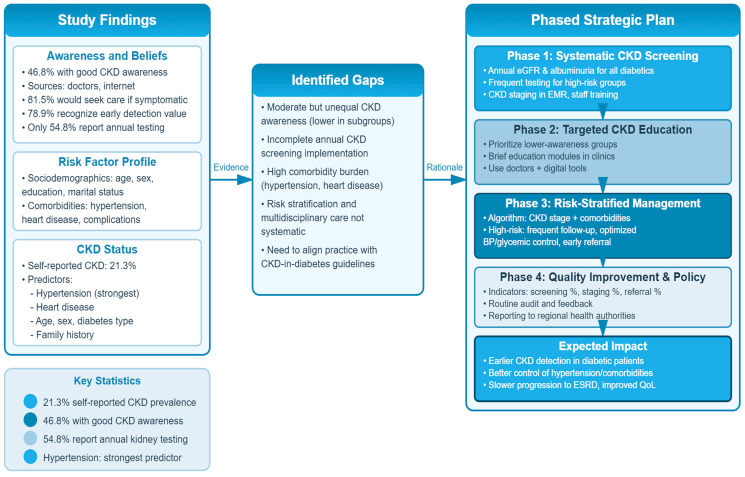
Conceptual framework and hypothetical phased strategy for CKD monitoring and management among patients with diabetes in the present region. CKD: chronic kidney disease; eGFR: estimated glomerular filtration rate; EMR: electronic medical record; BP: blood pressure; ESRD: end-stage renal disease; QoL: quality of life.

The conceptual framework and chart presented in this study (Figure 2) summarize these phases and pathways, linking our empirical findings (awareness level, risk factor profile, and screening gaps) to concrete components of a regional CKD monitoring and management strategy. Although this plan has not yet been implemented or evaluated, and should therefore be regarded as a hypothetical, conceptually driven framework rather than an empirically tested intervention, it provides a pragmatic basis for future intervention studies and for aligning local practice with international standards for diabetes-related CKD care.

## 5. Conclusions

This study shows that patients with diabetes in the Northern Border region of Saudi Arabia have a fair level of awareness of CKD, with unmarried men demonstrating more understanding. However, these findings are based on self-reported data and a non-probability sample, and both awareness and self-reported CKD prevalence estimates should be interpreted with caution. Hypertension and heart disease showed strong associations with self-reported CKD in this cohort, consistent with the established cardiometabolic risk profile of CKD, but the cross-sectional, self-reported design does not allow causal inferences. These findings underscore the urgent need for targeted educational interventions, routine evidence-based screening, and integrated management of comorbidities to reduce the burden of CKD among patients with diabetes. Enhancing patient education, promoting adherence to annual kidney-function assessment, and implementing structured risk-stratified monitoring pathways within diabetes care will be critical to improving outcomes and quality of life for this high-risk group in the Northern Border region and beyond.

## Figures and Tables

**Table 1:** Participants’ demographic characteristics (N = 389).

Demographic Information	Category	Frequency and Proportion *n* (%)
Age	18–25 years	203 (52.2%)
	26–35	92 (23.7%)
	36–46	66 (17.0%)
	More than 46 years	28 (7.2%)
Nationality	Saudi	371 (95.4%)
	Non-Saudi	18 (4.6%)
Sex	Female	303 (77.9%)
	Male	86 (22.1%)
Marital status	Single	191 (49.4%)
	Married	171 (44.0%)
	Divorced	20 (5.1%)
	Widow	6 (1.5%)
Education status	University or higher	259 (66.6%)
	Secondary school	94 (24.2%)
	Elementary	6 (1.5%)
	Intermediate	17 (4.4%)
	Illiterate	13 (3.3%)
The city	Arar	272 (69.9%)
	Rafha	10 (2.6%)
	Turaif	63 (16.2%)
	Other	44 (11.3%)

Data are presented in frequencies (*n*) and proportions (%).

**Table 2:** Medical data of the study participants (N = 389).

Questions	Categories	*n* (%)
What type of diabetes do you have?	Type 1 diabetes	124 (31.9%)
	Type 2 diabetes	45 (11.6%)
	I don’t know	220 (56.6%)
What is the duration of your diabetes diagnosis?	Less than 5 years	239 (61.4%)
	From 6 to 10 years	66 (17.0%)
	More than 10 years	84 (21.6%)
What type of treatment do you have for your diabetes?	Orally	217 (55.8%)
	Injection	99 (25.4%)
	Both of them	73 (18.8%)
Do you suffer from chronic kidney disease?	Yes	83 (21.3%)
	No	251 (64.5%)
	I don’t know	55 (14.1%)
How long have you been diagnosed with a chronic kidney condition?	Less than 5 years	278 (71.5%)
	From 6 to 10 years	41 (10.5%)
	More than 10 years	70 (18.0%)
Is there a history of kidney disease in your family?	Yes	104 (26.7%)
	No	285 (73.3%)
Do you suffer from other diseases?	Diabetic foot	11 (2.8%)
	Eye complications	18 (4.6%)
	Heart problems	20 (5.1%)
	High blood pressure	98 (25.2%)
	Neuropathy	16 (4.1%)
	Other	226 (58.1%)

Data are presented in frequencies (*n*) and proportions (%).

**Table 3:** Awareness of chronic kidney disease (CKD) among the study participants (N = 389).

Questions	Categories	*n* (%)
Do you know that chronic kidney disease reduces the kidneys’ ability to remove toxins from the bloodstream?	Yes	224 (62.7%)
	No	82 (21.1%)
	I don’t know	63 (16.2%)
Are you aware that diabetes can be a significant factor in the development of chronic kidney disease?	Yes	236 (60.7%)
	No	88 (22.6%)
	I don’t know	65 (16.7%)
Are you familiar with the signs and symptoms of chronic kidney disease?	Yes	180 (46.3%)
	No	128 (32.9%)
	I don’t know	81 (20.8%)
Did you know that, until the disease progresses to a more advanced stage, a patient with chronic kidney disease may not exhibit any symptoms?	Yes	191 (49.1%)
	No	109 (28.0%)
	I don’t know	89 (22.9%)
Did you know that elevated blood pressure can lead to chronic renal disease?	Yes	188 (48.3%)
	No	110 (28.3%)
	I don’t know	91 (23.4%)
Are you familiar with any complications of chronic kidney disease?	Yes	187 (48.1%)
	No	115 (29.6%)
	I don’t know	87 (22.4%)
Did you know that end-stage renal failure can develop from chronic kidney disease?	Yes	236 (60.7%)
	No	80 (20.6%)
	I don’t know	73 (18.8%)
Did you know that kidney failure can be fatal unless treated with dialysis or a kidney transplant?	Yes	247 (63.5%)
	No	66 (17.0%)
	I don’t know	76 (19.5%)
Do you know that treating kidney failure is more expensive than testing kidney function?	Yes	219 (56.3%)
	No	79 (20.3%)
	I don’t know	91 (23.4%)
What is your source of information about chronic kidney disease?	Doctor	154 (39.6%)
	Internet	103 (26.5%)
	Patients with CKD	28 (7.2%)
	Awareness message of the Ministry of Health	28 (7.2%)
	Others	76 (19.5%)

Data are presented in frequencies (*n*) and proportions (%).

**Table 4:** Opinions and beliefs about chronic kidney disease among the study participants (N = 389).

Statements	Categories	*n* (%)
Your blood sugar is under control and within a healthy range	Yes	327 (84.1%)
	No	62 (15.9%)
Your blood pressure is under control and within the healthy range	Yes	312 (80.2%)
	No	77 (19.8%)
Did you know that kidney function testing is necessary even if you do not have symptoms of chronic kidney disease?	Yes	275 (70.7%)
	No	114 (29.3%)
Do you undergo routine annual kidney function testing?	Yes	213 (54.8%)
	No	176 (45.2%)
Did you know that chronic kidney disease is a major life-threatening condition?	Yes	301 (77.4%)
	No	88 (22.6%)
If you had symptoms of kidney disease, would you visit a health center?	Yes	317 (81.5%)
	No	72 (18.5%)
Did you know that there are effective strategies to prevent chronic kidney disease?	Yes	292 (75.1%)
	No	97 (24.9%)
Do you know that early detection of chronic kidney disease is essential to slow its progression?	Yes	307 (78.9%)
	No	82 (21.1%)

Data are presented as frequencies (*n*) and proportions (%).

**Table 5:** The association between participants’ demographic and their awareness of chronic kidney disease (CKD).

Variables	Category	Awareness of CKD		*p*-Value
		Poor	Good	
Age	18–25 years	118 (58.1%)	85 (41.9%)	0.100
	26–35 years	49 (53.3%)	43 (46.7%)	
	36–46 years	28 (42.4%)	38 (57.6%)	
	More than 46 years	12 (42.9%)	16 (57.1%)	
Nationality	Saudi	198 (53.4%)	173 (46.6%)	0.780
	Non-Saudi	9 (50.0%)	9 (50.0%)	
Sex	Female	172 (56.8%)	131 (43.2%)	0.008 *
	Male	35 (40.7%)	51 (59.3%)	
Marital status	Single	113 (58.9%)	79 (41.1%)	0.009 *
	Married	79 (46.2%)	92 (53.8%)	
	Divorced	9 (45.0%)	11 (55.0%)	
	Widow	6 (100.0%)	0 (0.0%)	
Education status	University or higher	135 (52.1%)	124 (47.9%)	0.936
	Secondary school	51 (54.3%)	43 (45.7%)	
	Elementary	4 (66.7%)	2 (33.3%)	
	Intermediate	10 (58.8%)	7 (41.2%)	
	Illiterate	7 (53.8%)	6 (46.2%)	
The city	Arar	145 (53.3%)	127 (46.7%)	0.947
	Rafha	6 (60.0%)	4 (40.0%)	
	Turaif	32 (50.8%)	31 (49.2%)	
	Other	24 (54.5%)	20 (45.5%)	
Source of information about CKD	Doctor	46 (29.9%)	108 (70.1%)	<0.001 *
	Internet	70 (68.0%)	33 (32.0%)	
	Patient with CKD	15 (53.6%)	13 (46.4%)	
	Awareness message of the Ministry of Health	17 (60.7%)	11 (39.3%)	
	Others	59 (77.6%)	17 (22.4%)	

Data are presented as frequencies (*n*) and proportions (%). Chi-square (χ^2^) or Fisher’s exact test is applied. * Statistically significant level at *p* < 0.05.

**Table 6:** The correlation between participants’ attributes and the reported chronic kidney disease.

Variables	Self-Reported Prevalence of CKD			
	Category	No Chronic Kidney Disease	Chronic Kidney Disease	*p*-Value
Age	18–25 years	168 (82.8%)	35 (17.2%)	0.033 *
	26–35 years	68 (73.9%)	24 (26.1%)	
	36–46 years	53 (80.3%)	13 (19.7%)	
	More than 46 years	17 (60.7%)	11 (39.3%)	
Nationality	Saudi	294 (79.2%)	77 (20.8%)	0.203
	Non-Saudi	12 (66.7%)	6 (33.3%)	
Sex	Female	254 (83.8%)	49 (16.2%)	<0.001 *
	Male	52 (60.5%)	34 (39.5%)	
Period diagnosed with diabetes	Less than 5 years	190 (79.5%)	49 (20.5%)	0.806
	From 6 to 10 years	50 (75.8%)	16 (24.2%)	
	More than 10 years	66 (78.6%)	18 (21.4%)	
Type of treatment for your diabetes	Orally	163 (75.1%)	54 (24.9%)	0.141
	Injection	81 (81.8%)	18 (18.2%)	
	Both of them	62 (84.9%)	11 (15.1%)	
Type of diabetes	Type 1 diabetes	76 (61.3%)	48 (38.7%)	<0.001 *
	Type 2 diabetes	30 (66.7%)	15 (33.3%)	
	I don’t know	200 (90.9%)	20 (9.1%)	
Family history of kidney disease	Yes	51 (49.0%)	53 (51.0%)	<0.001 *
	No	255 (89.5%)	30 (10.5%)	
Comorbidity	Diabetic foot	8 (72.7%)	3 (27.3%)	<0.001 *
	Eye complications	14 (77.8%)	4 (22.2%)	
	Heart problems	13 (65.0%)	7 (35.0%)	
	High blood pressure	50 (51.0%)	48 (49.0%)	
	Neuropathy	14 (87.5%)	2 (12.5%)	
	Other	207 (91.6%)	19 (8.4%)	

Data are presented as frequencies (n) and proportions (%). Chi-square (χ^2^) or Fisher’s exact test is applied. * Statistically significant level at *p* < 0.05.

**Table 7:** Multivariate logistics of self-reported chronic kidney disease (CKD).

Factor	aOR	95% CI	*p*-Value
Age			
• More than 46 years	Ref		
• 18–25 years	0.66	0.310–1.405	0.281
• 26–35 years	1.03	0.469–2.243	0.951
• 36–46 years	0.65	0.252–1.673	0.371
Sex	0.64	0.339–1.198	0.162
Type of diabetes	0.33	0.175–0.637	<0.001 *
Family history of kidney disease	0.19	0.108–0.345	<0.001 *
Comorbidity			
• Other ^#^	Ref		
• Diabetic foot	1.79	0.390–8.233	0.453
• Eye complications	2.34	0.612–8.971	0.214
• Heart problems	4.21	1.351–13.125	0.013 *
• High blood pressure	5.77	3.117–10.673	<0.001 *
• Neuropathy	0.75	0.142–3.914	0.728

aOR—Adjusted Odds Ratio; CI—Confidence Interval. * Statistically significant level at *p* < 0.05. ^#^ ‘Other’ (absence of diabetic foot, eye complications, heart problems, hypertension, or neuropathy) was used as the reference category to represent participants without the specified major complications.

## Data Availability

The original contributions presented in this study are included in the article/Supplementary Material. Further inquiries can be directed to the corresponding author.

## Supplementary Materials

The following supporting information can be downloaded at https://www.mdpi.com/article/10.3390/diseases14020074/s1; the study questionnaire is in English.

## Abbreviations

The following abbreviations are used in this manuscript:

aOR	adjusted odds ratio
BP	blood pressure
CI	confidence interval
CKD	chronic kidney disease
eGFR	estimated glomerular filtration rate
EMR	electronic medical record
ESRD	end-stage renal disease
No	number
QoL	quality of life
