# Supplementary material for: Awareness and Perceived Risk Factors of Chronic Kidney Disease Among Patients with Diabetes in the Northern Borders of Saudi Arabia: Implications for a Strategic Monitoring and Management Plan
Source: Diseases. 2026 Feb 16;14(2):74. doi: 10.3390/diseases14020074 (PMC12939839; doi:10.3390/diseases14020074)
Supplement: Supplementary file 1 [file diseases-14-00074-s001.zip › diseases-4104078-supplementary.pdf]

Questionnaire in English

A-Personal information

1.	Age<br><br>18-25<br>26-35<br>36-46<br>More than 46
2.	Nationality<br><br>Saudi<br>Non-Saudi
3.	Sex<br><br>Male<br>Female
4.	Marital state<br><br>Married<br>Single<br>Divorced<br>widowed
5.	Educational status<br><br>University or higher<br>Secondary school<br>Preparatory school<br>Primary school<br>Illiterate
6.	City<br><br>Arar<br>Rafha<br>Turaif<br>Other

B- Medical data of the study participants

1.	What is your diabetes type?	Type 1<br>Type 2<br>I do not know
2.	How long have you been diagnosed with diabetes?	<5 years<br>6–10 years<br>>10 years
3.	What type of medications are you taking for the treatment of diabetes?	Oral<br>Injections<br>Both
4.	Are you suffering from chronic kidney disease (CKD)?	Yes<br>No<br>I do not know
5.	How long have you been diagnosed with chronic kidney disease?	<5 years<br>6–10 years<br>>10 years
6.	Is there a history of kidney disease in your family?	Yes<br>No
7.	Do you have any other medical conditions?	Hypertension<br>Heart complications<br>Neuropathy<br>Diabetic foot<br>Eye complications<br>others

C. Awareness of chronic kidney disease (CKD) among the study participants

1	Do you know that CKD reduces the kidneys' capacity to remove toxins from the bloodstream?	Yes<br>No<br>I don't know
2	Are you aware that diabetes can be a significant factor in the development of chronic kidney disease?	Yes<br>No<br>I don't know
3	Do you know any symptoms associated with chronic kidney disease?	Yes<br>No<br>I don't know
4	Do you know that CKD may not show any symptoms until it progresses to an advanced stage?	Yes<br>No<br>I don't know
5	Do you know that CKD can result from elevated blood pressure levels?	Yes<br>No<br>I don't know
6	Are you familiar with the complications of chronic kidney disease?	Yes<br>No<br>I don't know
7	Do you know that Chronic kidney disease can progress to end-stage renal failure?	Yes<br>No<br>I don't know
8	Do you know that kidney failure may result in death unless treated with dialysis or a kidney transplant?	Yes<br>No<br>I don't know
9	Do you know that treatment for kidney failure is	Yes

	more expensive than screening for kidney function?	No<br>I don't know
10	What is the source of your CKD information?	Doctor<br>Internet<br>Patient with CKD<br>Awareness message of MOH<br>Others

D. Opinions and beliefs about chronic kidney disease among the participants of the study

1	Your blood sugar is under control and within a healthy range	(yes, no)
2	Your blood pressure is under control and within a healthy range	(yes, no)
3	Do you know that a kidney function test is necessary, even if no CKD symptoms are apparent?	(yes, no)
4	Do you undergo the annual routine kidney screening test?	(yes, no)
5	Do you know that Chronic kidney disease represents a major risk of death?	(yes, no)
6	If you display symptoms of kidney disease, you will visit a healthcare center.	(yes, no)
7	Do you know that there are effective strategies to prevent chronic kidney disease?	(yes, no)
8	Do you know that early detection of CKD is essential to slowing down its progression?	(yes, no)
